# Diversification of the ruminant skull along an evolutionary line of least resistance

**DOI:** 10.1126/sciadv.ade8929

**Published:** 2023-03-01

**Authors:** Daniel P. Rhoda, Annat Haber, Kenneth D. Angielczyk

**Affiliations:** ^1^ Committee on Evolutionary Biology, University of Chicago, 1025 E. 57th St., Chicago, IL 60637, USA.; ^2^ Negaunee Integrative Research Center, Field Museum of Natural History, 1400 S. DuSable Lake Shore Dr., Chicago, IL 60605, USA.; ^3^ The Jackson Laboratory, Farmington, CT 06032, USA.

## Abstract

Clarifying how microevolutionary processes scale to macroevolutionary patterns is a fundamental goal in evolutionary biology, but these analyses, requiring comparative datasets of population-level variation, are limited. By analyzing a previously published dataset of 2859 ruminant crania, we find that variation within and between ruminant species is biased by a highly conserved mammalian-wide allometric pattern, CREA (craniofacial evolutionary allometry), where larger species have proportionally longer faces. Species with higher morphological integration and species more biased toward CREA have diverged farther from their ancestors, and Ruminantia as a clade diversified farther than expected in the direction of CREA. Our analyses indicate that CREA acts as an evolutionary “line of least resistance” and facilitates morphological diversification due to its alignment with the browser-grazer continuum. Together, our results demonstrate that constraints at the population level can produce highly directional patterns of phenotypic evolution at the macroevolutionary scale. Further research is needed to explore how CREA has been exploited in other mammalian clades.

## Teaser

A mammalian-wide pattern of allometry connects micro- to macro-evolution in the skulls of ruminant artiodactyls.

## INTRODUCTION

Natural selection acts on phenotypic variation in a population. Development structures variation and, consequently, the ways in which a population can respond to selection (*1*–*4*). The direction with the greatest amount of variation is termed the “line of least resistance” (LLR) (*5*–*7*). The LLR represents the direction of greatest potential for evolutionary change because it contains the most variation for selection to act upon and, presumably, because the biological processes underlying its bias easily accommodate evolutionary changes. Populations are expected to evolve in a direct path toward an adaptive peak if selection is aligned with the LLR, but the response to selection will be impeded and diverted toward the LLR if selection is oriented elsewhere (*1*, *8*, *9*). Therefore, the interaction between the adaptive landscape and constraints on variation within a species determines the trajectory of phenotypic evolution.

This interaction operates at the population level. Understanding how constraints within populations scale to explain global patterns of biodiversity is a fundamental goal in biological research, with the potential to elucidate the microevolutionary mechanisms producing macroevolutionary patterns. Simulations predict that, over macroevolutionary time scales, lineages with no constraints on phenotypic variation may explore all areas of morphospace uniformly, but a lineage with a highly constrained phenotype, where traits covary strongly (i.e., high phenotypic integration), will only explore areas of morphospace close to the LLR (*10*, *11*). As a result, highly constrained lineages have the potential to evolve more disparate phenotypes than would be expected under a Brownian motion model of evolution but only along the LLR. Empirically studying the macroevolutionary implications of intrinsic constraints is difficult because the adaptive landscape is dynamic, and population-level constraints themselves evolve (*12*). Accordingly, studying the relationship between conserved constraints and persistent sources of selection will help us understand the influence of population-level constraints on macroevolution.

**Fig. 1.**
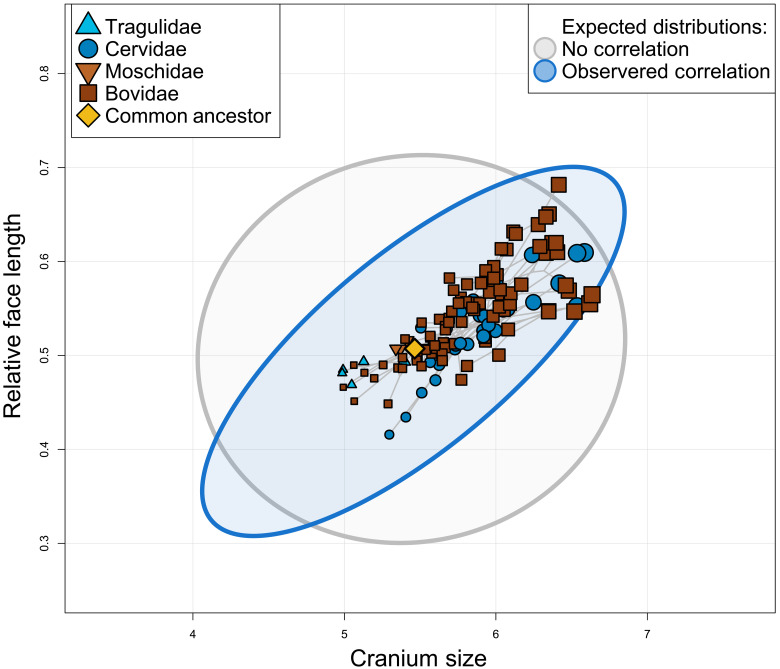
Larger ruminant species have proportionally longer faces, as predicted by CREA. The gray ellipse represents the 95% confidence interval of the expected distribution of species if there was no allometry of face length (given the observed evolutionary rates), and the blue ellipse represents the expected distribution of species given the observed evolutionary rate matrix. Note that certain “extreme” trait combinations (e.g., very large and long-faced species) are only accessible under correlated evolution, whereas other combinations (e.g., very small, long-faced species) are only accessible under hypothetical uncorrelated evolution.

In the mammalian skull, a highly conserved pattern of ontogenetic and evolutionary allometry is present, where larger individuals and species have proportionally longer faces [craniofacial evolutionary allometry (CREA); Fig. 1] (*13*–*15*). Allometry is historically thought of as a constraint on phenotypic evolution (*16*), and it is insofar as it makes certain trait combinations inaccessible, but allometry also presents an opportunity for extreme phenotypes to arise without developmental novelty:Extreme phenotypes can arise by “piggybacking” onto relatively labile evolutionary changes in size (*17*). In other words, larger, shorter-faced mammal species are improbable, but otherwise out-of-reach long-faced phenotypes are only possible in the largest mammals by exploiting this allometric pattern. CREA may therefore act as an evolutionary LLR and has been hypothesized as such (*14*, *15*, *18*, *19*).

In ruminant artiodactyls (deer, antelopes, goats, cattle, and relatives), size varies along an ecological axis separating smaller browsing species eating easily digestible foods such as fruit and larger grazing species subsisting on large amounts of low-growing, high-silica, and nutrient-poor vegetation [with exceptions; (*20*, *21*)]. Ruminant species span multiple orders of magnitude in body size, from the chevrotains and mouse deer in Tragulidae with masses between 2 and 3 kg to the gigantic Bovines reaching 1000 kg. Haber (*22*) found that macroevolutionary patterns of diversification in all ruminants were, in part, governed by population-level constraints on cranial shape and that, within bovids and cervids, species whose structure of variation was better aligned with their clade’s divergence have diverged farther away from their ancestor. A recent study on cranial diversification in Bovidae identified allometry (CREA) as the primary influence on large-scale evolutionary patterns (*23*). Here, we present strong evidence that variation in ruminants at the micro- and macroevolutionary levels is strongly biased by CREA, concordant with the definition of an evolutionary LLR, and demonstrate that exploitation of CREA facilitates morphological diversification in directions close to CREA.

## RESULTS AND DISCUSSION

### CREA governs macroevolutionary trends in the ruminant skull

A strong relationship between size and skull shape between species was found [*P* < 0.001, coefficient of determination (*R*^2^) = 0.196, *Z* = 5.927, *F* = 31.191], where larger species have longer faces (Fig. 1). The slope of evolutionary allometry varied between subfamilies significantly (*P* = 0.012), but, in each subfamily, larger species had longer faces (fig. S2).

**Fig. 2.**
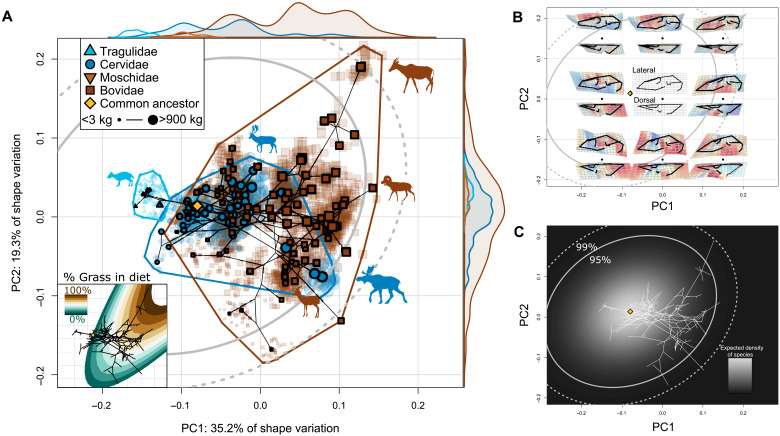
Evolutionary variation in the ruminant cranium is biased toward CREA. (**A**) Phylomorphospace of the species’ mean cranial shapes, with individual specimens projected back into the space. Gray ellipses denote 95 and 99% confidence intervals of the probability distribution of species in morphospace given a Brownian motion model of evolution. Size of data point (species) corresponds to centroid size. Distributions shown on the edges of the plot represent the relative densities of each family. Inset: An ecological surface highlighting variation in percent grass in diet, where grazing species consume primarily grass and browsing species consume less grass. Note that variation in percent grass in diet is similar to variation in species’ size. (**B**) Shape variation across the morphospace. Each black point represents a shape model at a given coordinate, with its lateral and dorsal views presented above and below, respectively. (**C**) Probability distribution of species in morphospace given our evolutionary rate matrix. Lighter areas denote more-likely areas a species will exist in morphospace given a Brownian motion model of evolution. An interactive version of this morphospace (and others) is available for nonmobile devices (https://danielrhoda.shinyapps.io/Ruminant_Dashboard/).

The major axes of interspecific variation preserve a clear trend in size (Fig. 2A), where larger species are located at the positive ends of principal component 1 (PC1) and PC2 (describing 35.2 and 19.2% of shape variation, respectively). The first PC captures variation in the relative length of the face and lateral expansion of the orbits (related to the characteristic tubular shape of bovid orbits). The second PC not only describes relative face length variation but also captures variation in the posterior retraction of the nasals relative to the premaxilla and overall slenderness of the skull (shapes closer to the positive end are more gracile with less nasal retraction and longer faces) (Fig. 2B). Distributions of the density of specimens (per family, accounting for different sample sizes of species; Fig. 2A) show a clear phylogenetic structure to PC1. The shapes of the species distributions are similar along PC2, but more diverse families extend farther in morphospace. Our estimated common ancestor is short-faced and small, observed at a negative PC1 score and near-zero PC2 score. Consequently, many of the ruminant species that extend beyond our 95% confidence intervals are large bovids with positive PC1 scores. Most notably, the hartebeest, *Alcelaphus buselaphus*, explores extreme PC1^+^ and PC2^+^ values and has the longest face of the species in our dataset. Most small ruminant species lie near the common ancestor’s shape, but the dik-diks (genus *Madoqua*) have more negative PC2 scores than any other ruminant. The cranial shapes of dik-diks are unlike the other small ruminants in having greater nasal retraction. Regardless, all small ruminants—the tragulids, moschids, and browsing cervids and bovids—have relatively shorter faces than their massive, long-faced relatives. Moose, *Alces americanus* and *Alces alces*, are the only cervids to extend beyond the 95% confidence interval of the interspecific morphospace and deviate far from all other cervids except *Megaloceros giganteus*, the Irish elk. Expectedly, *Alces* and Irish elk are by far the two largest cervids in our dataset. Our phylogenetic principal component analysis (pPCA) and phylogenetically aligned component analysis (PACA) morphospaces both show clear size trends dominating the major axes of variation, further strengthening our findings (figs. S3 to S5). Lineages that explore the most distant regions of morphospace are particularly short-faced and small or long-faced and large.

**Fig. 3.**
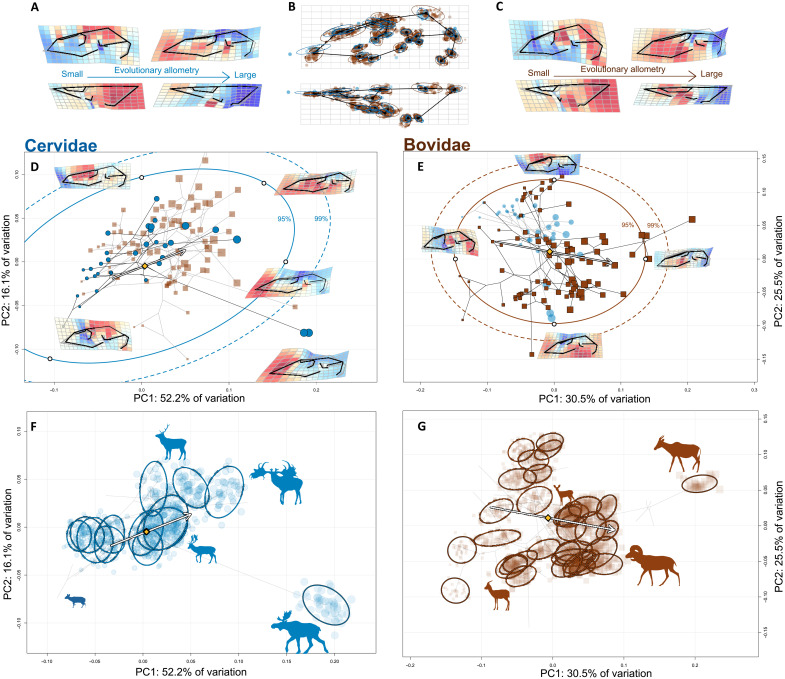
CREA governs phenotypic variation within both Cervidae and Bovidae. (**A** and **C**) Evolutionary allometric trajectories of the cervid (A) and bovid (C) cranium. (**B**) Mean shape of the dataset, with the Procrustes superimposed landmark scatters colored by family and scaled according to species’ centroid size. (**D** and **E**) Phylomorphospaces of Cervidae and Bovidae, with confidence intervals of the probability distribution of species in morphospace, as in Fig. 2. Arrows denote allometric trajectories, as in (F) and (G). (**F** and **G**) Family-specific morphospaces with individual specimens projected into them and ellipses for each species in the intraspecific dataset.

We recreated the interspecific morphospace for Cervidae and Bovidae individually to understand potential differences between the clades in their evolutionary patterns and exploitation of CREA (Fig. 3). Size explained large amounts of variation in both Cervidae (*P* < 0.001, *R*^2^ = 0.354, *Z* = 4.243, *F* = 17.506) and Bovidae (*P* < 0.001, *R*^2^ = 0.167, *Z* = 5.898, *F* = 17.287) (Fig. 3, A and C). A majority (52.2%) of cranial variation in the cervid skull is explained by the first PC, which describes the length of the face and nasal retraction (both increase toward positive end; Fig. 3, D and F). Other than *Alces*, interspecific differences in cervid skull shape are highly eccentric in the direction of CREA, but cervids occupy a restricted area of morphospace compared to Bovidae. We reconstructed this morphospace without *Alces* to see how cervids occupy morphospace without this divergent genus. The first PC of cervid morphospace without the moose is congruent with the allometric axis, and cervid populations are highly biased along this axis (fig. S6). The first PC of bovid morphospace describes variation in relative face length and height of the cranium and is well aligned with the family’s evolutionary allometric trajectory (Fig. 3, E and G). The second PC describes nasal retraction, with *Saiga* at the negative end and the frugivorous duikers (Cephalophini) at the positive end. The morphospace is notably similar to the morphospace of Bovidae presented by Bibi and Tyler (*23*) who argued that allometry was the primary influence on bovid skull diversification. We corroborate this finding. There is a clear size trend to the major axis of evolutionary variation in the bovid cranium, and it is this axis where the large, grazing tribe Alcelaphini and the small, browsing tribe Neotragini diversify farther than expected given their observed evolutionary rate. Within both families, patterns of intraspecific variation are biased in the directions of their allometric axis (Fig. 3, F and G), with exceptions (e.g., *Alces*, but note that the direction of *Alces*’ ellipse is congruent with the branch separating it from the rest of deer).

*Alces* is the farthest lineage along PC1^+^ in cervid morphospace by far, extending well beyond the 99% confidence interval. The saiga antelope, a bovid species with an unusually high degree of nasal retraction, is the only other taxon near *Alces*. Both *Saiga* and *Alces* have proboscises overhanging their mouths, but the adaptive significance of these structures seems to differ between the species (*24*, *25*). The *Saiga* lives in the deserts of the Central Asian steppe and uses its enlarged proboscis to regulate the entry of dust during respiration (*24*), but boreal moose often forage semiaquatically for sodium-rich vegetation that is required for antler growth and is absent in its terrestrial environment (*26*). It is hypothesized that moose use their proboscises as a valve to prevent the flow of water into the airway (*24*). Mating behaviors, either loud vocalizations amplified by the proboscis [*Saiga*; (*27*)] or “lip-curling” in *Alces* (*28*), serve as additional explanations for nasal retraction. The rest of cervid species and their constituent specimens are restricted to a scatter biased in the positive direction, highly congruent with the direction of CREA. It would be reasonable to assert that moose do not adhere to CREA because the branch leading to *Alces* is nearly orthogonal to CREA in this projection of tangent space, but this is partly misleading—*Alces* has the longest face and is the largest extant cervid genus, and the primary difference between it and other cervids is the unusual degree of nasal retraction. Notably, *Cervalces* (not present in our dataset), an extinct relative of *Alces*, does not share *Alces*’ peculiar nasal retraction (*29*) nor does the Irish elk, which is comparable in size to *Alces*. The modest size of *Saiga*, the presence of more nasally restricted morphologies in *Madoqua*, and the presumptive recent evolution of nasal restriction in *Alces* suggested by *Cervalces* all suggest that this morphotype is not necessarily related to size, but this relationship certainly requires additional research.

### The adaptive significance of CREA

The axis of shape variation associated with the browser-grazer continuum was significantly more aligned with CREA than two 75-dimensional vectors would be solely due to chance (angle = 73.96°, *P* = 0.0079), signaling similarity between these axes.

It is unclear whether the exceptionally long-faced crania of large grazing species like the Alcelaphini are (i) a direct result of selection for crania better adapted for grazing, which coincidentally happen to be long-faced and easily evolvable under the clade-wide allometric constraints, or are (ii) agnostic to selection for foraging ecology, with longer faces passively evolving during increases in body size associated with grazing (or decreases in size associated with browsing). The latter seems unlikely, considering that the face is intimately involved in food ingestion and processing. For example, a proportionally longer face would allow larger amounts of plant material to be processed at once and more room for cranial musculature to aid in mastication (*30*), which may be advantageous for large grazing species that eat gritty, fibrous materials. A longer face also keeps the eyes farther away from the ground, both protecting the eyes and facilitating better detection of predators (*31*–*32*). Regardless, as long as longer faces are not strictly maladaptive for grazing ecologies and shorter faces maladaptive for browsing, CREA would not obstruct evolution along the browser-grazer continuum and instead facilitate diversification of skull form. In either of our two probable scenarios, foraging ecology provides adaptive value for evolutionary changes in size and thus motivation for ruminant cranial morphology to diversify.

Although we have focused on the significance of a few exceptional lineages that defy the predictions of Brownian motion, most of the ruminant species have intermediate face lengths and sizes and cluster at neutral PC2 values. Most ruminants are not obligate browsers or grazers but are facultative “mixed feeders” who opportunistically forage on a combination of different plant materials. Multiple studies have suggested that lineages change their diets toward mixed feeding when faced with environmental turbulence (*33*–*35*). Central locations in interspecific morphospace (Fig. 2) may therefore be consistent with the concept of a “net adaptive peak” (*36*). In a biological system with functional trade-offs, the optimal phenotype (adaptive peak) is not necessarily one that maximizes a single function, but a neutral phenotype able to adequately perform multiple functions, with the relative importance of competing functions determined by the environment. The high density of cranial forms lying in central locations of morphospace, suitable for browsing and grazing, solidifies this claim. In Ruminantia, mixed feeding originated around the Oligocene-Miocene transition and triggered a period of taxonomic and ecological diversification (*37*). Grazing behavior emerged near the Mid-Miocene climatic optimum and triggered another radiation during the expansion of C_4_ grasses, but transitions between browsing and grazing (exclusively through mixed feeding) remained common (*37*). The data presented here suggest the evolutionary lability of ruminant diet through the Neogene, and therefore, their evolutionary success may, in part, be explained by the alignment of CREA and this ecological axis. As the environment changes, ruminant lineages can simply “slide” along an allometric axis to adapt to different relative amounts of browsing and grazing. Contrasting these observations in Ruminantia with perissodactyls may help explain perissodactyls’ declining diversity throughout the Cenozoic: Is the covariance structure of the perissodactyl skull less aligned with the browser-grazer continuum, obstructing ecological transitions and thus limiting diversification?

### Population-level variation is biased toward CREA

**Fig. 4.**
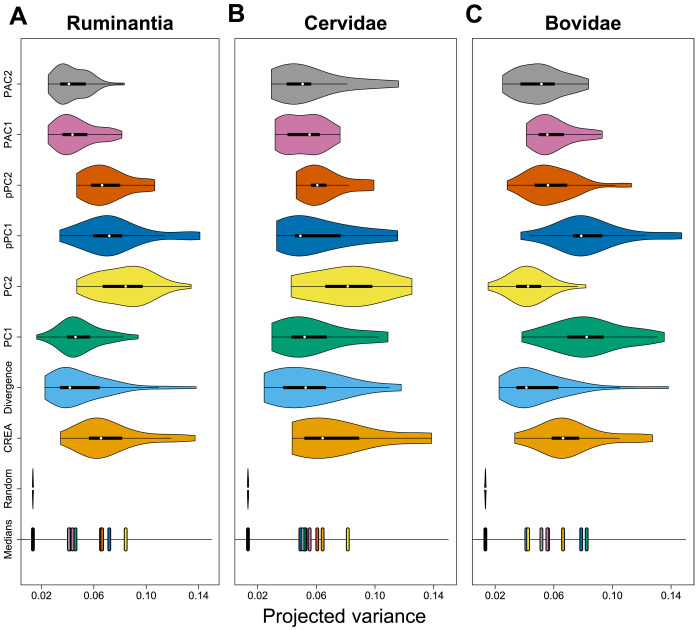
CREA explains as much intraspecific variation as the major axes of interspecific variation. Distributions of species’ projected variance values for different directions of evolutionary variation for all of Ruminantia (**A**), Bovidae (**B**), and Cervidae (**C**). Axes of evolutionary variation for Cervidae and Bovidae are calculated using only members of the respective clade. For example, PC1 and PC2 in the cervid and bovid panels are the axes presented in Fig. 3. The positions of medians of the distributions are at the bottom of each plot.

As discussed above, macroevolutionary patterns reveal a role for CREA in influencing morphological diversification, but how is this reflected at the population level in which selection operates? For CREA to qualify as an evolutionary LLR, intraspecific variation should be biased in the direction of CREA, species should diverge from their ancestor primarily in the direction of CREA, and species that align more closely with this direction should diverge farther. We find that intraspecific variation is consistently biased in the direction of CREA. CREA explained 3.5 to 13.7% of variation within species, which was much greater than the variation explained by the distribution of random vectors (Fig. 4A), suggesting that allometry is a bias on intraspecific variation. The major axes of evolutionary variation explained similar amounts of variation as CREA; only PC2 of the interspecific morphospace explained considerably more variation, indicating that intraspecific variation is as aligned with CREA as it is with the other major directions of evolutionary variation (Fig. 4A). Population-level variation was even more biased toward CREA than the direction in which the species diverged.

**Fig. 5.**
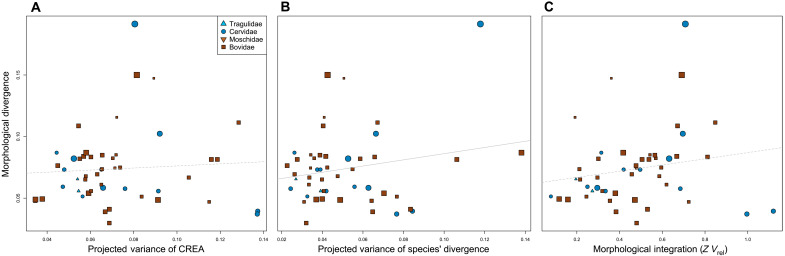
CREA and morphological integration facilitate morphological divergence. Scatterplots of properties of **P** and the magnitude of divergence from an ancestor. Each point represents a species, with the point size corresponding to centroid size. Dashedregression lines denote nonsignificant relationships. (**A**) Projected variance of CREA. (**B**) Projected variance of the direction of divergence. (**C**) Morphological integration effect size.

**Fig. 6.**
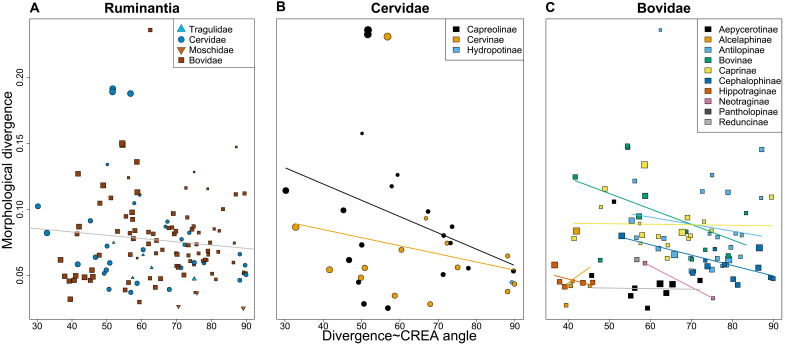
Species that diverged in the direction of CREA diverged farther than species that did not. Scatterplots of the magnitude of morphological divergence and the angle between the direction of divergence and CREA. All ruminants in our dataset (**A**), Cervidae (**B**), and Bovidae (**C**). Each point is a species, and point sizes correspond to centroid size.

Phylogenetic generalized least squares regressions found no significant relationship between the amount of variation within species in the direction of CREA (*V*_proj_ of CREA) and the magnitude of species’ divergence from their subfamily’s ancestor (*P* = 0.952, *F* = 0.003; Fig. 5A), but there was a significant relationship between the magnitude of divergence and the amount of variation in **P** explained by divergence (*P* = 0.034, *F* = 4.76; Fig. 5B). As mentioned above, species with their structure of variation aligned with CREA may already lie on adaptive peaks and have no incentive (or ecological opportunity) to diverge far. The lack of ruminant species with low *V*_proj_ values that have diverged far is consistent with our predictions. Species with higher morphological integration diverged farther from their ancestors as well (*P* = 0.123, *F* = 2.47; Fig. 5C). *Odocoileus virginianus* and *Odocoileus hemionus* were the most tightly integrated species and most biased toward CREA, but they did not diverge far from their ancestor. Their high *Z*_rel_ and *V*_proj_ values may be due to the presence of subspecies if the axis of variation separating subspecies is similar to the primary axes of variation within the subspecies. When removing *Odocoileus*, we find a strong positive relationship between integration and divergence (*P* = 0.011, *F* = 7.04) and a slightly stronger but nonsignificant relationship between bias toward CREA and divergence (*P* = 0.315, *F* = 1.03). The positive relationship between integration and divergence suggests that highly eccentric variation is contributing to morphological diversification, but that this eccentric variation is not always due to allometry. Species that diverged in the direction of CREA diverged farther than species diverging elsewhere (*P* = 0.012, *F* = 6.44) (Fig. 6A). This relationship is present in cervids but not significant (*P* = 0.087, *F* = 3.12) (Fig. 6B) and less so in bovids (*P* = 0.181, *F* = 1.82) (Fig. 6C). There seem to be certain ruminant subfamilies that preserve this relationship, such as Bovinae, Antilopinae, Cephalophinae (Fig. 6C), and the cervid subfamilies Capreolinae and Cervinae (Fig. 6B), but, of the six ruminant subfamilies with at least 10 species (the previously mentioned subfamilies and Caprinae), the relationship is only significant in Cephalophinae (*P* = 0.012, *F* = 9.18). Note that, in hyperdimensional spaces like the one here (*k* = 75), a distribution of random angles will be normally distributed around 90° with a lower standard deviation than lower-dimensional spaces (*38*). The angles of divergence and CREA were significantly different than a distribution of 10,000 random *k*-dimensional angles [two-sided Kolmogorov-Smirnov test, *P* < 0.001; (*67*)] (fig. S8), meaning that morphological divergence was more aligned with CREA than would be expected by chance.

### Micro- and macroevolutionary consequences of CREA as a LLR

Together, the relationships between the structure of variation (**P**) and the direction and magnitude of divergence reinforce the argument that CREA is being exploited as an evolutionary LLR. At the macroevolutionary level, the cranial morphology of ruminants diversified farther than expected under our observed evolutionary rates, assuming Brownian motion, but only in the direction of CREA. At both micro- and macroevolutionary scales, highly integrated cranial variation led to the exploration of greater ranges of morphospace. In any given system, there are likely numerous selective pressures in different directions, and any given structure of integration is impeding responses to some of these pressures and facilitating responses to others. A universal relationship between the strength of integration and disparity is therefore unlikely, and the relationship should depend on the relative strengths of the different selective pressures and their alignment with **P**’s within a clade of interest. In the case of Ruminantia, size dominates cranial variation through allometric scaling of facial length and is related to foraging ecology, signaling congruence between the direction of selection and integration. Accordingly, here, we generally find positive relationships between morphological integration and disparity at both micro- and macroevolutionary levels. If not for the alignment between a highly conserved allometric pattern and selection, then this positive relationship is unlikely.

Craniofacial elongation is characteristic of postnatal growth within mammalian species. Our analyses suggest that this well-known allometric mechanism, where the growth of the face outpaces the braincase, defines an evolutionary LLR and governs both population- and clade-level patterns of morphological diversity in Ruminantia. Haber (*22*) demonstrated that, in the ruminant skull evolutionary, divergence is well aligned with **P** for most species and that the better the alignment, the farther the species diverge from their clade’s ancestor. Our reanalysis of Haber’s landmark dataset expands these findings and implicates a mammalian-wide allometric pattern as the biological process likely underlying these consequential intrinsic constraints. There is still, however, substantial flexibility within the constraint imposed by CREA. The structure of **P** has evolved throughout the history of Ruminantia (*39*), possibly due to other factors influencing skull form such as head gear. Despite these reorganizations in covariance structure, CREA consistently explains as much or more intraspecific variation as the other major directions of evolutionary divergence (Fig. 4A). It is unrealistic to expect that covariance structure is perfectly conserved throughout a clade’s history, especially considering that variation in directions other than CREA needs to be maintained to respond to other selective pressures, but the analyses presented here demonstrate that certain biological processes at the population level can impose constraints that dominate macroevolutionary trends even as covariance structure evolves.

### CREA and mammalian evolution

Craniofacial elongation characterizes allometric patterns within and between mammalian species of nearly all placentals (*14*, *15*, *40*, *41*), probably marsupials (*42*, *43*), and some groups of nonmammalian synapsids [pelycosaurs, gorgonopsians, and possibly others; (*19*)]. The CREA pattern is highly conserved and deeply rooted in mammalian evolution. Does CREA act as an evolutionary LLR in other clades? And if so, how ubiquitous is exploitation of CREA in mammals? We speculate that exploitation of CREA is probably common or at least not rare, considering the many ways modifying relative length of the face can confer adaptive value. For example, a relatively shorter face generates stronger bite forces because of mandibular lever mechanics, which has consequences for feeding ecology. Phyllostomid bats contain the highest dietary diversity of any mammalian family and have crania more tightly integrated than their relatives (*44*). The major axis of cranial variation in Phyllostomidae distinguishes nectivorous and frugivorous species and almost exclusively describes variation in relative facial length, which, in turn, determines mechanical advantage of the cranium and the most suitable food types (*44*–*48*). Furthermore, facial length dominates evolutionary variation in clade-wise analyses of bats (*49*–*51*), and heterochrony has been invoked as a primary mechanism generating cranial diversity in the clade (*52*, *53*). The exploitation of CREA as a LLR seems likely in Chiroptera as in ruminants (*54*), and other mammalian clades may similarly deploy simple evolutionary changes in size to achieve adaptation of cranial form through CREA.

This study builds on a growing body of literature showcasing the profound macroevolutionary implications that ontogenetic allometric patterns may have [e.g., (*55*–*61*)]. The findings presented here suggest that morphological diversification of the ruminant skull proceeded along an evolutionary LLR defined by allometry (CREA) and that the eccentricity of population-level variation acted as a facilitator to morphological diversification in that direction because this direction of variation confers adaptive value. This study demonstrates that biases on intraspecific variation can be reflected in macroevolutionary-scale morphological trends, in this case over 30 million years of ruminant evolution. A key goal of future work should be to understand the ubiquity of exploitation of CREA as a LLR in mammalian evolution and to contrast the macroevolutionary consequences of CREA in clades where changes in relative facial length have different fitness consequences.

## MATERIALS AND METHODS

To quantitatively test hypotheses about evolutionary constraint, a comparative dataset of intraspecific variation is desirable. We analyzed a previously published dataset of 2859 ruminant crania from 130 species [(*22*); with the addition of *Saiga tatarica* and *M. giganteus*, the extinct Irish Elk; (*62*)] using geometric morphometric methods. The *Saiga* skull was gathered from MorphoSource (NMNH USNM 336264, ARK identifier: ark:/87602/m4/M100779). Landmark definitions are presented in the Supplementary Materials. All specimens are adults. We split Haber’s (*22*) landmark data into two datasets: an interspecific dataset, with a mean landmark configuration representing each species (*n* = 130, approximately 65% of extant ruminant species), and an intraspecific dataset including only the species represented by at least 27 specimens (*n* = 49) (*39*, *63*). Generalized Procrustes superimpositions were used to separate shape from scale, location, and orientation of the raw landmark data (*64*). Analyses were performed in a phylogenetic context when possible using a recent molecular phylogeny of mammals (*65*) pruned to our taxon sample. This phylogeny did not contain divergence time estimates for subspecies of *Odocoileus*, so we did not divide *O. hemionus* or *O. virginianus* into their subspecies as in (*22*, *39*). All analyses were performed in R v4.0.5, primarily using the packages geomorph, Morpho, mvMorph, and custom scripts available at Daniel Rhoda’s Github (https://github.com/danielrhoda/ruminant_allometry) (*66*–*68*).

### Macroevolution: Interspecific metrics and predictions

We estimated the direction of evolutionary allometry (CREA) with a phylogenetic regression of multivariate shape onto log-transformed centroid size in a phylogenetic context using the procD.pgls function in geomorph. We included a species’ subfamily as an interaction term in the model to test whether allometric slope significantly differed between subfamilies (fig. S2). Centroid size served as our body size proxy because it as available for all specimens and correlated very strongly with body mass estimates of a subset of our taxa (fig. S1) (*21*). To visualize patterns of evolutionary variation in the ruminant cranium, we ordinated our interspecific dataset, containing the mean cranium shape for each species, using PCA (Fig. 2). Each individual specimen was projected into this interspecific morphospace. We also ordinated the interspecific dataset both independent of phylogenetic signal (pPCA, with “GLS” and “transform” parameters as TRUE in the gm.prcomp function of geomorph) and aligned with phylogenetic signal [PACA; (*69*)] to investigate the relative importance of phylogenetic versus ecological signal in the dataset (figs. S3 to S5). An interactive dashboard was created to visualize the distribution of ruminant species in these different ordinations (https://danielrhoda.shinyapps.io/Ruminant_Dashboard/). To aid in the visualization of shape variation, we color-coded the cells of thin-plate spline (TPS) deformation grids according to how much larger or smaller (by area) cells are within the model compared to the reference (undistorted) TPS model. Two-dimensional representations of the three-dimensional morphology from the lateral and dorsal views are presented. Ruminantia is composed mainly of two families, Cervidae (deer) and Bovidae (cattle, antelopes, goats, and relatives), that are characterized by key differences in skull architecture, most notably the presence of annually shed antlers in male cervids but ever-growing horns in both sexes of bovids. In case these clades exploit CREA in different ways, we repeated the ordinations and the phylogenetic regressions of shape on log centroid size using only members of either Cervidae or Bovidae (Fig. 3), with the members of the excluded family projected back into the other’s morphospace.

To examine the relationship between cranial morphology and the browser-grazer continuum in ruminants, we collected percent grass in diet [a proxy for a species’ location on the browser-grazer continuum; (*32*)] from Codron *et al.* (*21*) for a subset of our interspecific dataset (*n* = 98). We identified the axis of shape variation corresponding to the browser-grazer continuum using phylogenetic regression and then measured the vector angle between this axis and CREA using the “angleTest” function in Morpho (*67*). We fit a second-degree polynomial surface to the empirical percent grass data and PC1 and PC2 scores (cumulatively describing 54.5% of total variation) using the surf.ls and trmat functions in the spatial R package (*70*). Exclusively sampling naturally occurring morphologies, rather than a grid of theoretical morphologies, has been shown to produce accurate surfaces (*71*). This ecological surface aids in the visualization of variation in diet as it relates to variation in morphology (Fig. 2 and fig. S7). The surface fit was significant (i.e., the empirical PC1/2 scores significantly covaried with the ecological data; *P* < 0.001).

In an integrated phenotype, accessible areas of morphospace are limited, but evolutionary rate is unaffected (*10*, *11*). Previous work (*10*, *11*) ask us to consider a “fly in a tube” model of the evolutionary consequences of integration, where a fly (phenotype) can “zip” around a tube (morphospace) at any speed (evolutionary rate) but only within a tube of a specific shape, dictated by integration pattern. Under Brownian motion, evolutionary rate is constant, and variance is proportional to time. Therefore, given an observed rate of phenotypic evolution, we can predict whether or not integration is facilitating or obstructing phenotypic evolution by testing whether disparity is proportional to rate (*11*). In some recent studies, this was tested by regressing per-landmark Procrustes variance onto evolutionary rate and observing the slope of the relationship [e.g., (*72*–*74*)]. In this study, our analytical units are species not landmarks, so we took a different approach to test the fly in a tube model. First, we fit PC1 to PC5 scores of the interspecific morphospace to a Brownian motion model of evolution to compute an evolutionary rate matrix of PC scores [function mvBM in mvMorph; (*68*)]. We could not compute a rate matrix for all PC axes because of computational constraints, so we chose to only include the “meaningful” PC axes sensu Bookstein (*67*, *75*). This rate matrix, **C**, contains the evolutionary rates of each PC along the diagonal and pairwise coevolutionary rates elsewhere. Assuming Brownian motion, **C** defines a multivariate normal probability distribution of species distribution in morphospace, centered at the estimated ancestral shape. In other words, if we were to simulate random walks of PC scores a large number of times from the ancestral shape given our observed evolutionary rate matrix, then the highest density of species would be concentrated around the ancestral shape with lower densities farther away from the ancestral shape (Fig. 2C). We calculated 95 and 99% confidence intervals for this probability distribution and overlayed it on our morphospaces to visualize areas in morphospace where lineages defy expectations. If CREA is being exploited as an LLR and facilitating diversification at the macroevolutionary level, then we would expect some lineages to evolve beyond the confidence intervals (i.e., “fly” to the ends of the “tube”) but only by following the trajectory defined by CREA.

### Microevolution: Intraspecific metrics and predictions

The questions that we address here are whether CREA is being exploited as an evolutionary LLR in ruminant artiodactyls and how this influences morphological diversification. Schluter (*7*) tested for the presence of an LLR by successfully predicting that the direction in which species diverge from their ancestors should be biased toward the principal direction of intraspecific variation [PC1 of an additive genetic covariance matrix, **G**, or a phenotypic covariance matrix, **P**, used as substitute for **G**; (*76*)] and that species with greater alignment between the direction of their variation and divergence should diverge farther. Statistically testing the congruence between evolutionary divergence and PC1 of **G** or **P** matrices has remained the standard means for documenting LLRs since Schluter’s seminal work [e.g., (*77*–*80*), although see (*81*)]. Considering PC1 as the de facto LLR is intuitive because, in the presence of a strong LLR, responses to selection should be channeled into the direction of the LLR, consequently forming the major axis of interspecific phenotypic variation. However, PC axes are the major axes of variation irrespective of any specific generative process, and their sensitivity to sample details means that there is no guarantee that a given axis will capture variation uniquely associated with a process of interest (*82*). A better approach, we contend, is to directly measure the axis of variation associated with a hypothesized LLR (e.g., some developmental process, in our case, CREA) and then interrogate its relationship with population-level and evolutionary variation. So as not to place undue importance on an arbitrary axis of variation, we adopted the “projected variance” approach used by Hunt (*83*) here. The **P** matrix for each species in the intraspecific dataset was projected onto the evolutionary allometric axis, described as a unit-length vector of allometric coefficients, using the following equation

$$V_{\mathrm{proj}}=x^{T}Px$$

where **P** is the species’ phenotypic variance-covariance matrix (of superimposed landmark data) and **x** is the vector of allometric coefficients (*T* indicates transpose). Projecting **P** onto **x** gives us a number, *V*_proj_, describing the proportion of shape variation in **P** described by the projected vector. A higher *V*_proj_ value indicates that the species’ variation is more biased in the direction of CREA. This measure is equivalent to the “evolvability” metric sensu Hansen and Houle (*84*) of a **P** matrix given a single direction. To examine how aligned population-level variation is with CREA versus other major axes of evolutionary variation (Fig. 4A), we compared the distribution of *V*_proj_ values from CREA to (i) a distribution of *V*_proj_ calculated from random vectors (for each species, the average of 499 random vectors), (ii) *V*_proj_ of the direction of divergence from the species’ subfamilies’ inferred ancestor, and (iii) the first two eigenvectors from alternative ordinations of the between-species covariance matrix (PCA, pPCA, and PACA). *V*_proj_ was also computed for bovids and cervids separately using their clade-specific ordinations and evolutionary allometric trajectories (Fig. 4, B and C).

If CREA is being exploited as an LLR, then species with **P** matrices closely aligned with CREA (higher *V*_proj_) should have diverged farther from their ancestor than less-aligned species, and species that have diverged in the direction of CREA should have diverged farther from their ancestor than species diversifying in other directions. It is also possible that species with strongly biased **P** matrices may already lie on an adaptive peak and are under stabilizing selection, so as long as species with marginal bias toward CREA have not diverged farther from their inferred ancestor than species with strong bias, CREA may have been exploited as an LLR. We tested whether the direction of species’ divergence was biased by CREA by comparing the magnitude of a species’ divergence from its inferred ancestor to (i) the species *V*_proj_ value of CREA, the species *V*_proj_ of the direction of its divergence (the amount of variation a population has in the direction it evolved from), and magnitude of morphological integration (Fig. 5C); and (ii) the angle between the direction of divergence and direction of CREA (Fig. 6). Morphological integration was quantified as the standardized effect size of relative eigenvalue variance of each species’ **P** [*Z*_rel_, integration.Vrel function in geomorph; (*63*, *85*)], which has been shown to perform better than other integration metrics under controlled simulations (*86*, *87*). Morphological divergence was calculated as the Euclidean distance between the PC scores of a species and the PC scores of its subfamily’s inferred ancestor [as in (*22*)]. The most recent common ancestor of the subfamily was used rather than the position of the immediate ancestral node because our dataset does not contain all ruminant species, and the immediate ancestral node’s position is reliant on only the positions of its two descendants. The angle between divergence and CREA was computed by calculating the difference in coordinate values between a species’ landmark configuration and that of its subfamily’s inferred ancestor and then by taking the angle between this vector and the vector of evolutionary allometric coefficients. Phylogenetic generalized least-square regressions (*88*, *89*) were used to measure the associations between morphological divergence and the angle of divergence relative to CREA as well as between morphological divergence and integration and *V*_proj_.

## Notes

View/request a protocol for this paper from *Bio-protocol*.
